# Role of Polyamines in Immune Cell Functions

**DOI:** 10.3390/medsci6010022

**Published:** 2018-03-08

**Authors:** Rebecca S. Hesterberg, John L. Cleveland, Pearlie K. Epling-Burnette

**Affiliations:** 1 University of South Florida Cancer Biology Graduate Program, University of South Florida, 4202 East Fowler Ave, Tampa, FL 33620, USA; Rebecca.Hesterberg@moffitt.org; 2 Department Immunology, PharmD, Moffitt Cancer Center & Research Institute, 12902 Magnolia Drive, 23033 SRB, Tampa, FL 33612, USA; 3 Department of Tumor Biology, Moffitt Cancer Center & Research Institute, 12902 Magnolia Drive, Tampa, FL 33612, USA; John.Cleveland@moffitt.org

**Keywords:** immunity, T-lymphocytes, B-lymphocytes, tumor immunity, metabolism, epigenetics, autoimmunity

## Abstract

The immune system is remarkably responsive to a myriad of invading microorganisms and provides continuous surveillance against tissue damage and developing tumor cells. To achieve these diverse functions, multiple soluble and cellular components must react in an orchestrated cascade of events to control the specificity, magnitude and persistence of the immune response. Numerous catabolic and anabolic processes are involved in this process, and prominent roles for l-arginine and l-glutamine catabolism have been described, as these amino acids serve as precursors of nitric oxide, creatine, agmatine, tricarboxylic acid cycle intermediates, nucleotides and other amino acids, as well as for ornithine, which is used to synthesize putrescine and the polyamines spermidine and spermine. Polyamines have several purported roles and high levels of polyamines are manifest in tumor cells as well in autoreactive B- and T-cells in autoimmune diseases. In the tumor microenvironment, l-arginine catabolism by both tumor cells and suppressive myeloid cells is known to dampen cytotoxic T-cell functions suggesting there might be links between polyamines and T-cell suppression. Here, we review studies suggesting roles of polyamines in normal immune cell function and highlight their connections to autoimmunity and anti-tumor immune cell function.

## 1. Introduction

Metabolic regulation is a vital component of a coordinated immune response [1]. Dormant immune cells circulate in blood and tissues and morph into highly activated cells following antigen exposure. Activated immune cells act as sentinels throughout the body, and eradicate pathogens present in distinct ecosystems, in areas with diverse growth factors or low oxygen [2], and when nutrients are limiting [3], which can compromise their functional veracity. For a versatile and potent response, immune cells must make rapid and precise adaptations to these environmental changes [4]. To achieve its diverse functions, the immune system is comprised of heterogeneous populations of cells that are each capable of a broad range of responses. Importantly, all of these cells must adjust their metabolic activity to meet functional demands that include migration, proliferation and sometimes long-lasting persistence in these diverse environments [5,6].

Recent advances in understanding immunometabolism have shown that the energetic demands of unique T-cell subpopulations are linked to dynamic responses of the immune system. Most immune cells generate adenosine triphosphate (ATP) from glucose as their primary energy source, but drastic changes in metabolism are observed when transitioning from a quiescent to an activated state [7,8] and the complexity of metabolic circuits has confounded ascribing a particular function to one specific pathway or intermediate. Here, a focused discussion is provided that reviews the roles of an understudied metabolic pathway in immune cells, specifically that which controls polyamine homeostasis, in normal immune cell functions and immune-related diseases [9].

## 2. B-Cell Lymphopoiesis and Activation

As members of the adaptive immune system, T- and B-lymphocytes are fundamental components of an integrated immune response [10]. B-cell differentiation starts in the fetal liver and continues in the bone marrow during adult life [11]. Though both B- and T-cell populations are derived from a common lymphoid progenitor (CLP) in bone marrow [11,12,13,14], T-cells and B-cells differ by their mechanism of antigen recognition [15]. Specifically, B-cells express surface immunoglobulin (Ig) as a receptor for detecting circulating microorganisms. Antigen binding to Ig receptors activates B-cells and triggers their differentiation into plasma cells that produce and secrete copious amounts of soluble antibodies with distinct isotypes that selectively bind to the activating antigen. Further, a subset of antigen-activated B-cells differentiate into long-lasting memory B-cells, which allow for a more rapid response following re-exposure to cognate antigen [16].

The initial step in activating a B-cell response involves receptor-antigen interactions that occur in restricted areas of primary lymphoid organs such as the spleen, lymph node or tonsils [16]. On its surface, each B-cell expresses a single membrane bound Ig receptor (B-cell receptor, BCR) that is created through a unique process of somatic genomic recombination of immunoglobulin genes to form heterodimeric immunoglobulin receptors that results from the fusion of three separate gene segments, variable (V), diversity (D) and joining (J) genes (VDJ) that provide receptor diversity [15,17]. Both integrated T-cell and innate immune cell interactions are required for the activation of B-cells, which become progressively more antigen reactive via a process of hypermutation and class switching [16,18,19,20]. Precursor, immature and mature B-cells signal through the immunoglobulin receptor. Immature B-cells, expressing only membrane IgM heavy chain (mu) and the Ig_α_ and Ig_β_, [21] undergo several selection events triggered by the recognition of self-molecules in bone marrow that prevent autoimmunity [9]. Since the V(D)J-BCR gene rearrangement process is stochastic, there is a random expression of self-reactive receptors that requires a systematic bioenergetic reprogramming to achieve clonal deletion or inactivation of self-reactive B-cells in circulation [18,22]. Autoreactive B-cells have been shown to increase glycolysis and oxygen consumption compared to normal antigen-activated B-cells [22,23]. Further, disabling glycolysis by treatment with the pyruvate dehydrogenase inhibitor dichloroacetate impairs antibody production both ex vivo and in vivo [22]. Moreover, B-cell specific deletion of the glucose transporter Glut1 or Myc revealed their role in B-lymphopoiesis, and that c-Myc is necessary for activation-induced expression of Glut1 [22,24]. Notably, overexpression and inhibitor studies have revealed that c-Myc directly and coordinately induces the transcription of ornithine decarboxylase (ODC), adenosylmethionine decarboxylase-1 (Amd1), spermidine synthase (Srm), and spermine synthase (Sms), four enzymes which direct polyamine biosynthesis [25]. Indeed, c-Myc itself is a transcription factor for ODC and Sms [26,27]. Ornithine decarboxylase functions as a dimer and is the rate-limiting enzyme in the pathway and converts ornithine to putrescine, which is then converted into spermidine and spermine. Ornithine decarboxylase is tightly controlled by rapid messenger RNA (mRNA) turnover, a very short protein half-life, as well as by antizyme that is translationally induced as polyamine levels rise and which directly binds to ODC and triggers its destruction by the proteasome [28]. Gene knockout studies in mice have established that ODC is essential for proper embryogenesis [29].

Increased expression of enzymes that direct polyamine production and polyamine levels occur after BCR activation [30]. Further, addition of spermine compromises activation-associated apoptosis, suggesting polyamines may be important in repressing the clonal deletion of B-cells after activation. Moreover, nitric oxide enhanced IgE class-switching by anti-trinitrophenyl (TNP) keyhole limpet hemocyanin-(KLH) is blocked in vivo by treatment with aminoguanidine, which inhibits serum diamine oxidase and prevents the conversion of extracellular polyamines into toxic products [31,32]. Thus, although there are scant reports directly linking polyamines to specific B-cell functions, the importance of Myc and the role that Myc plays in B-cell activation and development suggests direct links to polyamines.

## 3. The Role of Polyamines in T-Lymphopoiesis

T-cells express either an αβ or γδ T-cell receptor (TCR) that rearrange through non-homologous recombination of the V(D)J genes mediated by the activations of the recombination activating genes (*Rag*)1 and *Rag*2 [33,34] as described for B-cells [35]. Deletion of *Rag*1 or *Rag*2, whose expression is restricted to lymphocytes, leads to small lymphoid organs and to the complete loss of mature circulating T and B-cells in mice. Unlike B-cell development that largely occurs in the bone marrow, T-cells arise from a common lymphoid progenitor that migrates into the thymus [36,37] where environmental interactions with thymic epithelial cells [38], signaling via NOTCH1 [39,40] and TCR repertoire selection occurs at the population level through positive and negative selection processes similar to B-cells [33,34,35,41]. Most T-cells (95%) in the lymphoid compartment express αβTCRs [42], but are further delineated by surface expression of CD4 or CD8, which are required for major histocompatibility cluster (MHC)-class II and MHC-class I co-ligation, respectively [43,44]. The αβTCR receptor is also expressed on regulatory T-cells [45], on a minor population of natural killer (NK) T-cells [46], and on subtypes of intestinal intraepithelial lymphocytes (IELs) [47], which play regulatory roles in response to mucosal infections [48].

**Figure 1:**
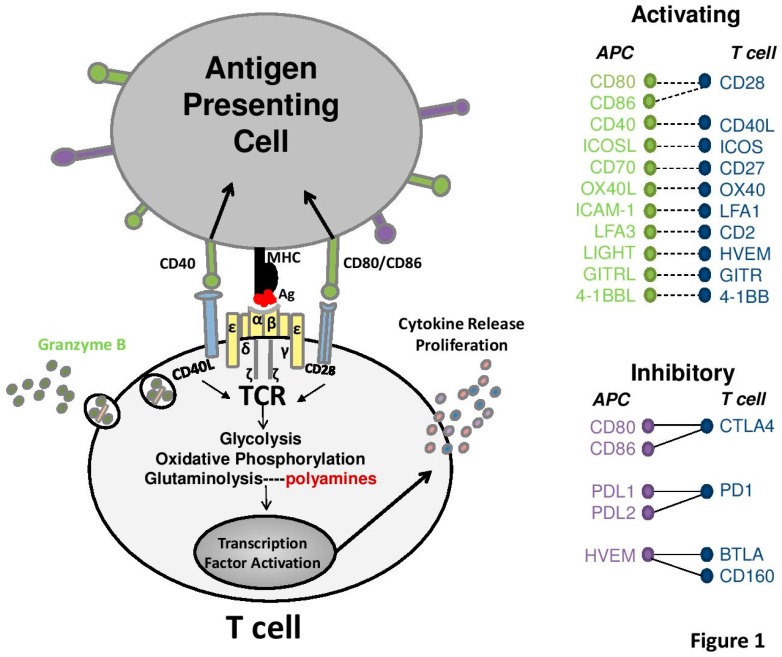
Stimulatory and inhibitory molecules expressed on T-cells. Diagram depicting the antigen presenting cell (APC) and T-cell interactions and activating receptors and ligands on these cells that govern the functional outcomes of T-cells such as cytotoxicity-associated granzyme B expression, cytokine release, and proliferation [61]. The T-cell receptor complex is composed of several proteins that are necessary for survival and signaling including T-cell receptor (TCR)α and TCRβ chains, CD3 signaling molecules δ/ε, CD3γ/ε and CD247 composed on the dimeric ζζ-chains or ζn (not shown). Co-stimulatory molecules on T-cells such as CD28, the founding member of the immunoglobulin (Ig) family of costimulatory receptors, are critical to amplify and sustain the signaling response. Activation leads to metabolic reprogramming to increase glycolysis, oxidative phosphorylation, and amino acid metabolism through glutaminolysis and ultimately to polyamine biosynthesis [62,63]. Additional receptors include CD40L (CD154), T-cell specific surface glycoprotein CD28, inducible T-cell costimulatory ICOS (CD278) which is a CD28-family molecule expressed on T-cells important for Th2 responses, Traf-linked tumor necrosis factor receptor family protein, CD27, which is important in T and B-cell memory formation and activation of natural killer (NK) cells [64,65,66], tumor necrosis factor receptor superfamily (TNFRSF), member 4 (TNFRSF4) also OX40 (CD134) expressed on activated T-cells [51,52,53], leukocyte-associated antigen-1 (LFA1) which is an integrin involved in T-cell migration [53,67], the adhesion molecule CD2 present on T-cells and NK cells (also known erythrocyte receptor and rosette receptor, LFA-2), herpesvirus entry mediator (HVEM) also known as tumor necrosis factor receptor superfamily member 14 (TNFRSF14), glucocorticoid-induced TNFR family related gene (GITR) a member of the TNFRSF [68], S-type lectin Galectin 9, T-cell immunoglobulin mucin domain 1 (TIM1) also known as hepatitis A virus cellular receptor 1 (HAVcr-1), and 4-1BB (CD137, TNFRS9). Corresponding receptors on APC are the classical costimulatory ligands CD80 (B7-1), CD86 (B7-2) that interact with CD28, TNFRS5 (CD40), human inducible costimulatory-ligand (ICOSL) [69], ligand for CD27 (CD70 also TNFSF7), OX40 ligand (OX40L), intercellular adhesion molecule 1 (ICAM-1, also CD54), leukocyte-associated antigen-3 (LFA3), HVEM counter-receptor lymphotoxin-like, exhibits inducible expression, and competes with herpes simplex virus glycoprotein D for HVEM, a receptor expressed by T-lymphocytes (LIGHT, also CD160), GITR ligand (GITRL), and 4-1BB ligand (4-1BBL). Inhibitory receptors and ligands are shown including cytotoxic T-lymphocyte antigen-4 (CTLA4) which interacts with CD80, CD86 that also recognizes CD28, programmed cell death protein 1 (PD1) receptor and its ligands PD-ligand 1 (PDL1) and PD-ligand 2 (PDL2), and B-and T-lymphocyte attenuator (BTLA) and CD160 [70] that both recognize HVEM. MHC: major histocompatibility complex, Ag: antigenic peptide.

A major difference between B- and T-cells is the MHC-restricted nature of TCR antigen activation [43]. T-cells recognize their targets (e.g., virally infected cells) through interaction of small peptide fragments bound in the groove of an MHC molecule, which strengthens selectivity for self over non-self and protects against autoimmunity [43,44]. Professional antigen-presenting cells (APCs) such as B-cells, macrophages and dendritic cells (DC) express both MHC class I and MHC class II for activating CD4^+^ and CD8^+^ T-cells. Through receptor or phagocytosis-mediated antigen internalization, APCs process antigen into the correct fragment length for display by the MHC molecule [49]. These cells also express additional co-stimulatory signals including CD28, OX40 ligand, CD40L (Figure 1), which enhances the T-cell’s response and provides a critical level of regulation [50,51,52,53,54]. Notably, the inducible co-stimulatory (ICOS) molecule, a member of the CD28 family, is essential for the T-cell mediated induction of immunoglobulin isotype class switching by activated B-cells [19]. Further T-cells undergo an educational process in the thymus mediated by Aire, a transcription factor expressed by medullary epithelial cells (mTECs) in the thymus, which induces the promiscuous expression of restricted peripheral tissue antigens (PTAs) [55] that trigger the clonal deletion of T-cells with potential self-reactivity before they can exit the thymus [56,57]. In part, this is due to the unique ability of Aire to recognize the hypomethylated amino-terminal tail of histone H3 [38,58], to bind to transcriptional sites of paused polymerases [59], and to control genes that direct mRNA splicing [57]. This process is critical to the formation of immunological tolerance, autoimmune prevention, and antitumor immunity [56,57,59,60].

**Figure 2:**
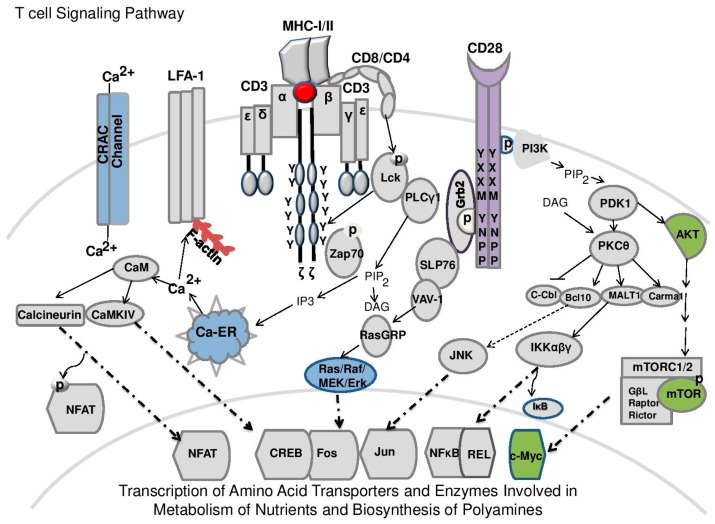
Proximal T-cell signaling cascade. Proximal signaling pathways downstream of the T-cell receptor (TCR)-antigen presenting cell (APC)ignaling complex (as described in Figure 1) are responsible for the cascade of events leading to metabolic reprogramming including the transcription of amino acid transporter and enzymes involved in metabolism of nutrients and biosynthesis of polyamines [85]. Phosphorylation of the immunoreceptor tyrosine-based activation motifs (ITAMs) on the cytoplasmic side of the TCR/CD3 complex engage numerous cascading interactions largely mediated by phosphorylation, dephosphorylation or ubiquitinylation resulting in cellular activation [61]. The initiating signal is generated by lymphocyte protein tyrosine kinase (Lck) and other proto-oncogene tyrosine-protein kinase (Src) family tyrosine kinases including the zeta-chain associated protein kinase (Zap-70) that is recruited to the TCR/CD3 complex. Costimulation through leukocyte-associated antigen-1 (LFA1) which is an integrin involved in T-cell migration or CD28 interaction with CD80 (B7-1) or CD86 (B7-2) (see also Figure 1) activates the phosphorylation of the YXXM or YNPP signaling motifs [86] which regulates glucose metabolism. CD28 leads to stable recruitment of the adaptor protein Grb2/GADS along with interleukin-2-indicible T-cell kinase (Itk), Lck, and phosphatidylinositide 3 kinase (PI3K) heterodimer p85/p110 and SLP76. These interactions promote the activation of VAV-1, RasGRP, and the Ras/Raf/MEK/Erk pathway downstream of phosphorylated SLP-76 and Zap-70 modulating the TCR signal strength [86]. A complement of transcription factors nuclear factor of activated T-cells (NFAT), cAMP response element-binding protein (CREB), Fox family transcription factor c-Fos, Jun (when in combination with c-Fos forms the AP-1 early response transcription factor complex, nuclear factor kappa-light-chain-enhancer of activated B cells (NFκB), an NFκB family member c-Rel, and c-Myc which coordinately regulate gene expression. Activation of CD28 leads to the phosphorylation of PI3K, phosphatidylinositol-3,4 bisphosphate (PIP2) and phosphoinositide-dependent kinase 1 (PDK1) [87] which integrates the TCR and CD28 signaling to induce the NFκB pathway including protein kinase C-theta (PKC-θ), and inhibits the ubiquitin ligase c-Cbl [88] leading to activation of Bcl10, Malt1, Carma1 (CBM) complex leading to IKKαβγ activation of NFκB and REL [87]. In addition to PKC-θ, phosphorylation of Akt is critical for the regulation of mTORC1 and mTORC2 complexes of mTOR that bind GβL and raptor or rictor, respectively [79,81]. This is a critical step in c-Myc-dependent transcriptional regulation that stimulates dramatic changes in metabolism including glucose, amino acid, nucleotide and polyamine biosynthesis [63,89]. Divalent cations such as calcium (Ca^2+^) are induce downstream of phospholipase C γ1, PIP2, and indo inositol-1,4,5 triphosphate (IP3) which mobilizes the release of intracellular Ca^2+^ stores from the endoplasmic reticulum (Ca^2+^-ER) a potential metabolic switch that suppresses intratumoral T-cell function [90]. Sustained signaling then promotes the influx of extracellular Ca^2+^ into the cells through calcium release-activated Ca^2+^ (CRAC) channels. Calcium-calmodulin interactions (Ca^2+^/CaM) then activates the phosphatase calcineurin and calcium/calmodulin-dependent protein kinase type IV calmodulin (CaMKIV), which dephosphorylates the cytoplasmic subunits of nuclear factor of activated T-cells (NFAT) exposing a nuclear localization signal resulting in nuclear transport and phosphorylates CREB, respectively.

Once released from the thymus, antigen-naive T-cells are primarily reliant on interleukin-7 (IL-7) which is critical for their growth and survival [71]. IL-7 directs the metabolic function of naïve cells by regulating basal glucose and amino acid metabolism via activation of Janus kinase (JAK3) and phosphorylation of signal transducer and activator of transcription-5 (STAT5) and PI3K/Akt/mTOR that promotes the surface expression of Glut1 and transport of glucose [72,73,74]. T-cells interact with peptide-loaded APCs in peripheral lymphoid organs, such as the spleen and lymph nodes, which stimulates the activation of their effector functions. Activation then triggers a complex cascade of signaling events (Figure 2) that leads to changes in metabolism [4,5,6]. Based on the cytokine milieu, CD4^+^ effector cells can differentiate into distinct subsets including T helper (Th)1, Th2, Th17, as well as FoxP3+ CD4^+^ regulatory T-cells (Tregs) which are all metabolically distinct [7,75]. Differential regulation of mammalian target of rapamycin mTOR, protein kinase B(Akt)-mediated phosphorylation of the tuberous sclerosis complex (TSC1/TSC2), and Ras family GTPase Rheb are critical in regulating this process [76,77,78,79]. Most notably, suppression of TOR complex 1 (mTORC1) pharmacologically and through genetic depletion of mTOR in T-cells leads to a predominance of Treg differentiation [80]. Functional specificity of mTOR is determined by its interacting proteins. The mTORC1 complex contains a small GTPase Rheb, a regulatory-associated protein of mTOR (raptor), the G protein β-subunit-like protein (GβL, also known as mLST8) and substrate 40 kDa (PRAS40) whereas, mTORC2 contains mTOR, and GβL with the rapamycin-insensitive companion of mTOR (rictor) and mammalian stress-activated protein kinase interacting protein-1 (mSin1) [81]. Signaling events such as activated AMP-activated protein kinase (AMPK) [82,83] that differentially antagonize the activation of mTORC1, polarize T-cell differentiation toward Tregs and simulate lipid oxidation [23]. Several surface markers such as l-selectin (CD62L) are also critical for metabolic reprogramming since they regulate homing and migration of T-cells into and out of lymphoid organs [84]. Although they express classical αβTCRs, NKT-cells function independent of MHC class I or II via interactions with a glycolipid antigen in the context of CD1d, a non-canonical MHC molecule. Based on the current literature, several of these fundamental events appear controlled by polyamines and/or are linked to key signaling molecules like mTOR or Myc (Figure 2) that control polyamine homeostasis.

Required role of polyamines in proper erythrocyte differentiation have been shown in studies with alpha-difluoromethylornithine (DFMO), a suicide inhibitor of ODC [91,92], but the impact of polyamines on lymphocyte development is largely unknown. Given established roles for putrescine (1,4-diaminobutane), spermidine and spermine in cell proliferation, DNA and RNA synthesis [93,94], as well as in protein translation in both cell free systems and in activated lymphocytes [62], polyamines are highly likely to play key roles in T-cell or B-cell development, particularly in scenarios where exogenous polyamines are limiting and there is compensatory mechanisms induced by polyamine uptake through designated energy-dependent transporters [95,96,97,98,99,100,101].

Conditional gene targeting in T-cells is accomplished using the lymphocyte-specific protein tyrosine kinase Lck or CD4-gene promoter fused Cre recombinases [39,102]. Expression of genes under the control of the *Lck* proximal promoter initiates conditional inactivation of genes early in T-cell development prior to the expression of T-cell lineage markers [103] versus CD4-Cre which directs gene expression after transition from the CD4^+^/CD8^+^ double-positive cell leading to gene deletion in both mature CD4^+^ and CD8^+^ single lineage T-cells in the periphery [39,43]. Although cell-specific *Odc* deletion in T-cells or B-cells has yet to be reported, several studies have assessed the effects of regulators of the polyamine pathway. The mTOR serine/threonine protein kinase senses the nutrient state and exists as two distinct protein complexes, mTORC1 and mTORC2. Cell growth (mass) is regulated by mTORC2 via c-Myc and, in turn, c-Myc coordinately induces polyamine biosynthetic enzymes through direct transcriptional regulation and through other mechanisms of regulation [26,27,63]. Notably, T-cells lacking *c-Myc* in *LckCre; c*-*Myc*^fl/fl^ mice are severely defective in their proliferative response and fail to undergo progression through the double positive (CD4+/CD8+) stage, which is likely due to failed proliferation by early pre-TCR signaling [104]. Further, deletion of Mnt, a Myc antagonist, triggers apoptosis of thymic T-cells and blocks T-cell development [105]. As a target of Myc [25], select depletion of *Odc* in T-cells is needed to assess the importance of polyamines on thymic development.

## 4. Role of Polyamines in Antigen Activated T-Cells

Given that ODC enzymatic activity is significantly increased after T-cell activation, polyamine production is an important part of normal T-cell function [82,92,93]. Though other ODC-regulating proteins have been reported, c-Myc is the major regulator of enzymes involved in polyamine biosynthesis in T-cells [25,87]. Indeed, mice deficient in another transcriptional regulator of ODC, c-Fos, have been shown to have normal peripheral T-cells, further demonstrating that c-Myc is the master regulator of T-cell-associated polyamines [106,107].

Two of the amino acid precursors for ornithine, glutamine and arginine, are required for T-cell activation [108,109] downstream of TCR signaling events, including mTOR, Myc and mitogen-activated protein kinases/extracellular signal-regulated kinases (MAPK/ERK) [63,109] that are linked through integrated signaling (Figure 2). Polyamines are likely produced downstream of either arginine or glutamine due to the increase in ODC enzymatic activity [63,110,111]. Mass spectrometry-based global metabolomics and integrated transcriptome analyses have been used to map the changes in metabolic intermediates after TCR-stimulation [112]. Notably, proteins that regulate the arginine and proline pathways are enriched in TCR-stimulated CD4^+^ T-cells, and metabolic tracing studies have shown that TCR activation triggers flux of L-arginine Arg into ornithine, putrescine, and agmatine, and to lower levels of spermidine and proline. Catabolism of Arg into polyamines in CD4^+^ T-cells is regulated by mitochondrial arginase-2 (ARG2) as arginase-1 is not expressed in these cells. Interestingly, dietary supplementation of Arg during activation is associated with enhance mitochondrial oxidative phosphorylation (OXPHOS) and mitochondrial spare respiratory capacity (SRC) [113,114,115]. The morphology and numbers of mitochondria are critical determinants for SRC and in T-cells, for a functional memory response following secondary antigenic challenge [113,114,115]. Notably, in vivo Arg supplementation of transgenic mice bearing a TCR receptor that specifically recognizes the hemagglutinin antigen (HA 110–119 peptide) increases intracellular Arg levels and the survival of memory T-cells [112].

Although polyamines have not yet been shown to be involved in the memory response, the role of polyamines in survival in other cells suggests that proper polyamine pools may be necessary for this response [25,116,117]. Further, similar to phenotypes observed in other cell types, polyamines are required for T-cell proliferation manifest after TCR stimulation [63,118]. Accordingly, though the mechanism (s) is unclear, polyamine depletion during initial T-cell activation in vitro has been shown to impair cytotoxic function (CTL) against target cells [119,120,121,122,123,124].

## 5. Role of Polyamines and Anti-Tumor Immunity

**Figure 3:**
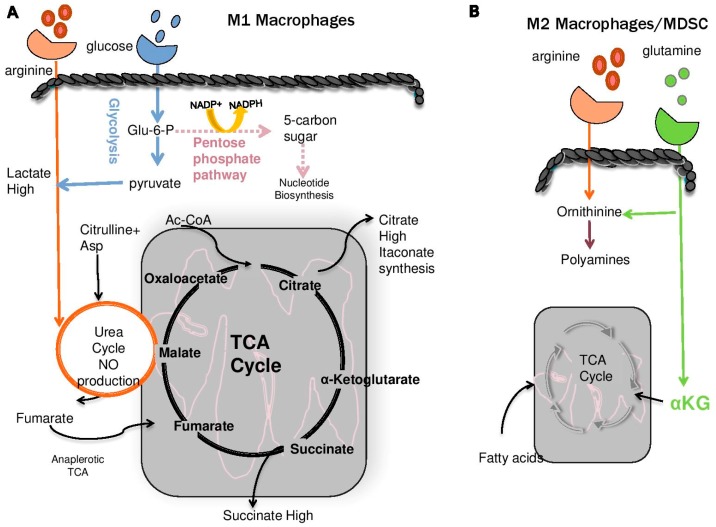

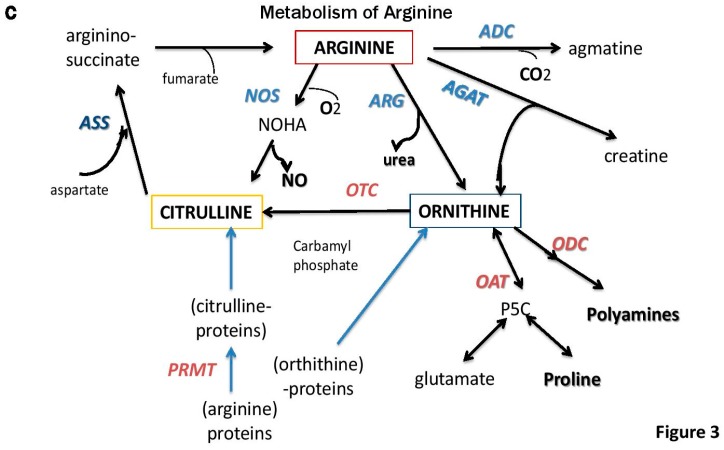
Bioenergetics of macrophage subsets. Monocyte-derived macrophages can be differentially polarized by the cytokine milieu [125,126]. (**A**) M1 macrophages originate from cells in the bone marrow and develop in inflammatory environments. Nitric oxide (NO) is the major byproduct of these cells arising from the reaction of arginine with oxygen through the actions of inducible nitric oxide synthase (iNOS) which produces citrulline and NO (see detailed pathway Figure 3C). Citrulline is then exported and re-imported to re-generate arginine and sustain NO production. A product of the degradation of arginine through this cycle is fumarate which is derived from the conversion of argininosuccinate to arginine (see Figure 3C). M1 macrophages are also critical for the production of cytokines and chemokines and for the production of itaconate which acts as an anti-microbial cellular metabolite. Succinate, a proinflammatory molecule that controls IL-1β expression, accumulates and stabilizes the oxygen sensing pathway regulated by hyposia-inducible factor 1-alpha (HIF1α) [125,127]. (**B**) Unlike M1 macrophages, the polarized M2 subtype reduces their ability to make NO and instead hydrolyzes imported arginine into ornithine and urea through the urea cycle (detailes in Figure 3C). M2 macrophages are therefore suppressive by competing for both arginine and glutamine that is necessary for effector T-cell functions [63,89,112]. To fuel their functions, including proliferation, M2 macrophages use fatty acids oxidation (FAO) which supports oxidative phosphorylation and electron transport through the tricarboxylic acid (TCA) cycle. Also present in the suppressive tumor microenvironment is a population of bone marrow derived immature myeloid cells known as myeloid derived suppressor cells [128,129]. While bioenergetics for these cells needs further analysis, they retain NO production and FAO, TCA and deplete arginine and glutamine [130] from the microenvironment. (**C**) Also detailed is the metabolism of arginine, l-citrulline and l-ornithine to produce fumarate from conversion of argininosuccinate. Citrulline plus aspartate generates argininosuccinate via the actions of argininosuccinate synthetase (ASS) in the cytosol and ornithine is converted to citrulline by carbamylphosphate plus ornithine via the enzymatic activity of ornithine transcarbamoylase (OTC). Additional enzymes and reactions include those metabolized by ODC: ornithinine decarboxylase, ARG: arginase 1 or arginase 2, ADC: arginine decarboxylase which is the biosynthetic enzyme for agmatate [131], OAT: ornithine aminotransferase, NOS: nitric oxide synthase, PRMT: protein arginine methyltransferases which is important for epigenetic regulation [132], and AGAT: l-arginine:glycine amidinotransferase which is the enzyme that catalyzes the transfer of an amidino group from l-arginine to produce l-ornithine and guanidinoacetate and acts as the immediate precursor of creatine.

Polyamines are essential components of T-cell and B-cell activation, where for example they are necessary for the effector functions and high rates of proliferation of T-cells [63,119,120,121,122,123,124]. However, polyamines play much different roles in other cell types of the immune system (Figure 3).

Surprisingly, several studies have demonstrated that ODC inhibition [133,134,135,136], and/or treatment with polyamine transport inhibitors (PTIs) significantly reduces rates of tumor growth and that this is due to increase in anti-tumor immunity. Further, the anti-tumor response is linked to T-cell anti-tumor activity, as the beneficial effects observed following treatment with ODC inhibitors and PTIs are reversed in Rag^−/−^ mice lacking both T and B-cells, and in athymic nude mice that lack only T-cells consistent with activation of T-cells after polyamine depletion in tumor models [134,137]. Moreover, polyamine inhibition increases CD8^+^ T-cell infiltration into the tumor bed [116,134,137]. Though CD8^+^ T-cells isolated from a similar B16F10 melanoma model lack cytotoxic functions in vitro [136], it is clear that systemic polyamine inhibition of tumor-bearing mice restores T-cell anti-tumor immunity.

In the tumor microenvironment, cell populations suppress the immune response and contribute to tumor escape from immune surveillance [138]. These cells also use polyamines to invoke their suppressive activations and to support their metabolism (Figure 3). Suppressive myeloid cells are evident in many infectious diseases, including leishmaniasis [139], toxoplasmosis [140], candidiasis [141], and human immunodeficiency virus (HIV)-infected individuals [142], and are significantly elevated in tumor-bearing animals [128,143]. Comparable suppressive cells have been identified in both mouse models and human cancers including melanoma, breast cancer, pancreatic, non-small cell lung and leukemia [143].

Suppressive myeloid cells, specifically myeloid-derived suppressor cells (MDSCs), monocyte-derived M2 macrophages and some dendritic cells (DCs), can be present in high numbers in the tumor microenvironment. Based on the cytokine milieu, monocyte-derived macrophages can be polarized into M1 or M2 macrophages [116,144,145]. M2 macrophages do not make nitric oxide (NO), a major byproduct of M1 macrophages, and use arginase to hydrolyze imported arginine into ornithine and urea which depletes arginine in the tumor microenvironment, compromising intratumoral T-cell functions and survival [129,143,146]. Myeloid-derived suppressor cells (MDSCs) retain the ability to produce NO and high levels of reactive oxygen species (ROS) leading to nitration of tyrosine residues of the TCR which disrupts its interaction with the peptide-MHC complex during antigen presentation [49] (Figure 1). The suppressive functions of M2 macrophages relies on higher basal mitochondrial oxygen consumption rates driven by fatty acid oxidation (FAO) [147] and, accordingly, the development of M2 macrophages is blocked by inhibiting mitochondrial OXPHOS and FAO (Figure 3). Further, unlike M1 macrophages, M2 macrophages require glutaminolysis for proliferation and ODC inhibition through difluoromethylornithine (DFMO) or polyamine transport inhibitor treatment of tumor-bearing mice significantly reduces intratumoral suppressive MDSCs [116,134,137] which should improve Arg availability for T-cells that is necessary for their proliferation and persistence [108,112,148,149]. Polyamine inhibition also increases TNFα and IL-1 cytokine production by tumor infiltrating macrophages, suggesting reprogramming of macrophages into the M1 phenotype that augments presentation of tumor-associated antigens, increases citrulline export and import, and further supports the TCA cycle through arginine-derived fumarate [116,136]. Recently, it has been shown that arginine-derived polyamines produced by DCs induce IDO1 expression within the cell through Src kinase, which results in a more immunosuppressive phenotype [150]. This can also be exacerbated by bystander MDSCs that provide more polyamines in the extracellular milieu freely available to DCs. Inhibition of ODC by DFMO reduces this signaling network and promotes DCs to an immune stimulatory phenotype [150]. Thus, it appears that although polyamines are required for normal CD8^+^ T-cell functions, the net effects of polyamine depletion on suppressive myeloid cells is to increase anti-tumor CD8^+^ T-cell activity by restoring a more conducive tumor microenvironment.

## 6. Polyamines in Autoimmune Disease

Autoimmune diseases are provoked by abnormal, unchecked immune responses against normal host tissue, and are driven self-reactive TCRs and BCRs in the thymus and bone marrow. Further, suppressive immune populations including myeloid cells, regulatory T-cells (Tregs) and IELs, are necessary to establish peripheral tolerance against self-reactive effector T- and B-cells that escape negative selection [6,151,152]. Autoimmunity can arise in almost every peripheral tissue in the body, for example multiple sclerosis in the brain, thyroiditis and Graves’s disease in the thyroid, rheumatoid arthritis and ankylosing spondylitis in the joints, psoriasis, eczema and scleroderma in the skin, diabetes in the pancreas, and celiac disease, ulcerative colitis, and Crohn’s Disease that occur in the intestine. Interestingly, circulating polyamine levels are increased in patients with autoimmune diseases [153,154], polyamines have the ability to form nuclear aggregates [155,156,157] and it has been suggested that nuclear polyamine aggregates interact with DNA, RNA, or other macro-molecular structures to stabilize autoantigens. Strikingly, the most common autoimmune B-cell responses are generated to macromolecules such as double stranded DNA or single stranded DNA [158,159]. Abnormal polyamine structures have been noted in patients with systemic lupus erythematosus (SLE), and rheumatoid arthritis that are characterized by anti-nuclear antibodies consistent with this hypothesis.

## 7. Concluding Remarks

Recent studies have provided key mechanistic insights into how polyamines may regulate cell fate and proliferation. First, it has been shown that decreasing polyamine pools with the ODC inhibitor DFMO reduces pools of the methyl donor S-adenosylmethionine (SAM, an activated form of methionine) [160]. This appears to occur via effects of polyamines on harnessing the translation of SAM decarboxylase (SAMDC/AMD1) [161,162], which converts SAM to decarboxylated SAM (dcSAM) [163]. Thus, reductions in polyamine pools lead to increases in dcSAM and corresponding reductions in SAM pools. Notably, methylation of DNA and histone tails requires the transfer of the methyl group derived from SAM, and these epigenetic changes are required for changing the pattern of peripheral tissue antigens during negative selection [38,60]. Furthermore, unbiased metabolomic analyses of colon tumor cells revealed that treatment with DFMO also leads to profound reductions in thymidine and thymidine monophosphate (TMP), and that inhibitory effects of DFMO on growth can be overcome by treatment with exogenous thymidine [160,161]. Collectively, these findings suggest a model whereby BCR- and TCR-dependent activation of c-Myc coordinately induces polyamine biosynthesis, and where polyamines then regulate B-cell and T-cell growth, fate, and effector functions via both epigenetic and metabolic control.
